# Fatty alcohols production by oleaginous yeast

**DOI:** 10.1007/s10295-015-1674-x

**Published:** 2015-08-29

**Authors:** Sandy Fillet, Jordi Gibert, Beatriz Suárez, Armando Lara, Carmen Ronchel, José L. Adrio

**Affiliations:** Neol Biosolutions SA, Avda. Innovación, 1, Edificio BIC-213, Parque Tecnológico de La Salud, 18016 Granada, Spain

**Keywords:** Fatty alcohols, Metabolic engineering, Fatty acyl-CoA reductase, *Rhodosporidium toruloides*

## Abstract

We have engineered *Rhodosporidium toruloides* to produce fatty alcohols by expressing a fatty acyl-CoA reductase from *Marinobacter aquaeolei* VT8. Production of fatty alcohols in flasks was achieved in different fermentation media at titers ranging from 0.2 to 2 g/L. In many of the conditions tested, more than 80 % of fatty alcohols were secreted into the cultivation broth. Through fed-batch fermentation in 7 L bioreactors, over 8 g/L of C_16_–C_18_ fatty alcohols were produced using sucrose as the substrate. This is the highest titer ever reported on microbial production of fatty alcohols to date.

**Electronic supplementary material:**

The online version of this article (doi:10.1007/s10295-015-1674-x) contains supplementary material, which is available to authorized users.

## Introduction

Fatty alcohols (or long-chain alcohols) are important raw materials and consumer products, and can be widely used in the chemical industry to produce detergents, lubricants, plastics and cosmetics [4, 24]. The global market for fatty alcohols reached $5.2 billion in 2011 and is expected to grow at 4 % CAGR for the next decade [22]. Currently, fatty alcohols are produced via catalytic hydrogenation of fatty acids produced from natural oil and fat sources, primary coconut, palm, palm kernel, tallow and lard, and also by chemical hydration of α-olefins [7]. There are some environmental concerns about the current use of these raw materials. On one hand, the use of vegetable oils, mainly palm oils, could lead to deforestation issues, whereas the dependence on petroleum has raised concerns about greenhouse effects and climate change [11].

Enzymatic production of medium-/long-chain alcohols (C_8_–C_18_) is achieved by the reduction of different acyl-CoA molecules to the corresponding primary alcohols. These enzymes are typically referred to as fatty acid acyl-CoA reductases (FARs). FARs can be divided into “alcohol-forming” and “aldehyde-forming” classes [23]. The former catalyze a four-electron reduction of active forms of fatty acids to fatty alcohols, whereas the latter catalyzes a two-electron reduction of active forms of fatty acids to fatty aldehydes. Alcohol-forming FARs have been identified in plants, marine bacteria, mammals, birds, nematodes and humans [8, 10, 36]. Many aldehyde-forming FARs have been described in bacteria and cyanobacteria [30, 32].

Microbial production of fatty alcohols has been successfully achieved in *Escherichia coli* and *Saccharomyces cerevisiae* via metabolic engineering [26, 28, 32, 33, 36, 38]. In *S. cerevisiae*, a production of 100 mg/L using glucose, galactose or raffinose as carbon source was achieved [28, 33]. In *E. coli*, engineering approaches based on the expression of heterologous fatty acyl-CoA reductases, or expression of endogenous aldehyde reductases, together with the manipulation of structural genes in fatty acid metabolism led to strains able to produce 1.65 g/L C_12_–C_14_ alcohols [35] and over 1.7 g/L of C_12_–C_18_ fatty alcohols in fed-batch reactors [20]. The productivity was 28.3 mg fatty alcohols/g glucose. Very recently, engineering of alternative pathways to increase the acyl-coA pool and expression of a fatty acyl-CoA reductase from *Marinobacter aquaeolei* in *E. coli* improved C_14_–C_18_ fatty alcohol titer up to 3.5 g/L using glucose as carbon source [14].

Using yeast as host to produce fatty alcohols can be advantageous, since as they emerge from the FAS complex, newly synthesized fatty acyl-CoA can be directly used as substrates for fatty acyl-CoA reductases to make fatty alcohols. However, reported titers in *S. cerevisiae* are lower than those in *E. coli*, reaching 0.1–0.5 g/L [28, 33]. Interestingly, manipulation of not only the structural genes, but also the regulatory genes in *S. cerevisiae* lipid metabolism has been recently used to produce over 1.1 g/L 1-hexadecanol through fed-batch fermentation using resting cells [12].

Oleaginous microorganisms are considered attractive next-generation host candidates for the production of fatty acid-derived chemicals, as these species have the ability to provide large amounts of fatty acids or lipids as precursors [1, 25, 36].

*Rhodosporidium toruloides* is an oleaginous yeast that can be cultured to extremely high cell density (>100 g/L dry cell mass) and accumulate more than 60 % biomass as triglycerides [2, 37]. Its genome has been sequenced [39], and efficient genetic transformation and targeted gene deletion methods have been recently reported [17, 21]. Besides, this microorganism is able to metabolize different carbon sources including xylose [13, 18].

Here we reported, for the first time, the production of fatty alcohols by *R. toruloides*. Using as host an improved strain able to make over 60 g/L of oil (triglycerides) from lignocellulosic sugars [six, Gibert et al., manuscript in preparation] we cloned and successfully expressed an acyl-CoA reductase from *M. aquaeolei* VT8. Cultivation of one selected clone, using a carbon source, led to the production of over 8 g/L of C_16_–C_18_ fatty alcohols in fed-batch bioreactors. This is the highest titer ever reported to date.

## Materials and methods

### Strains, media and cultivations

The yeast strain used in this study was *R. toruloides* CECT13085, an oil-overproducing strain [6]. *Agrobacterium tumefaciens* AGL-1 strain was used to perform transformation assays in *R. toruloides*, while *E. coli* DH5α was used for all plasmid constructions.

*Escherichia coli* strains were grown at 37 °C in LB medium supplemented with ampicillin (10 mg/L) or kanamycin (30 mg/L). *R. toruloides* strains were grown at 30 °C in YPD medium (yeast extract 10 g/L, glucose 20 g/L, peptone 20 g/L).

Fatty alcohol production in flasks was checked in 500 mL flasks containing 100 mL of YPD medium, YPD4 medium (yeast extract 10 g/L, peptone 20 g/L, glucose 40 g/L), YPS4 medium (yeast extract 10 g/L, peptone 20 g/L, sucrose 40 g/L), YPF4 medium (yeast extract 10 g/L, peptone 20 g/L, fructose 40 g/L), S4 M medium (KH_2_PO_4_ 0.75 g/L, NH_4_NO_3_ 0.28 g/L, CaCl_2_·2H_2_O 0.4 g/L, MgSO_4_·7H_2_O 0.4 g/L, yeast extract 1.5 g/L sucrose 40 g/L), or DXM (glucose 70 g/L, xylose 40 g/L, corn steep liquor 9.6 g/L, acetic acid 4.5 g/L, formic acid 0.4 g/L, furfural 0.15 g/L). Flasks were cultivated at 30 °C and 250 rpm for 36 h.

Peptone and yeast extract were purchased from Conda (Spain). Corn steep liquor was purchased from Dadelos (Spain) and sucrose from Azucarera (Spain). Glucose, xylose, fructose, acetic acid, formic acid and furfural were from Sigma (Sigma Chemical, Spain).

### Plasmids

The plasmids used in this work are listed in Table 1.

**Table 1:** Plasmids used in this work

Plasmids	Characteristics	Reference
pGEM-T Easy	Ap^R^; ori^pUC^; lacZ´ (cloning vector)	Promega
pUR5750	Km^R^; p^gdpA^::*hph*::t^trpC^ flanked by the left and right borders of T-DNA	Dr. S. Gutierrez
pNEOL57	Km^R^; pUR5750 derivative containing an MCS	This work
pNEOL79	Ap^R^; pGEMT derivative containing an maqRt cassette and a G418Rt cassette.	This work
pNEOL102	Km^R^, pNEOL57 derivative containing an maqRt cassette and a G418Rt cassette	This work
pEX-K4–maqRt	pEX-K4 derivative containing gen *maqRt*	This work

### Construction of maqRt expression cassette

**Fig. 1:**
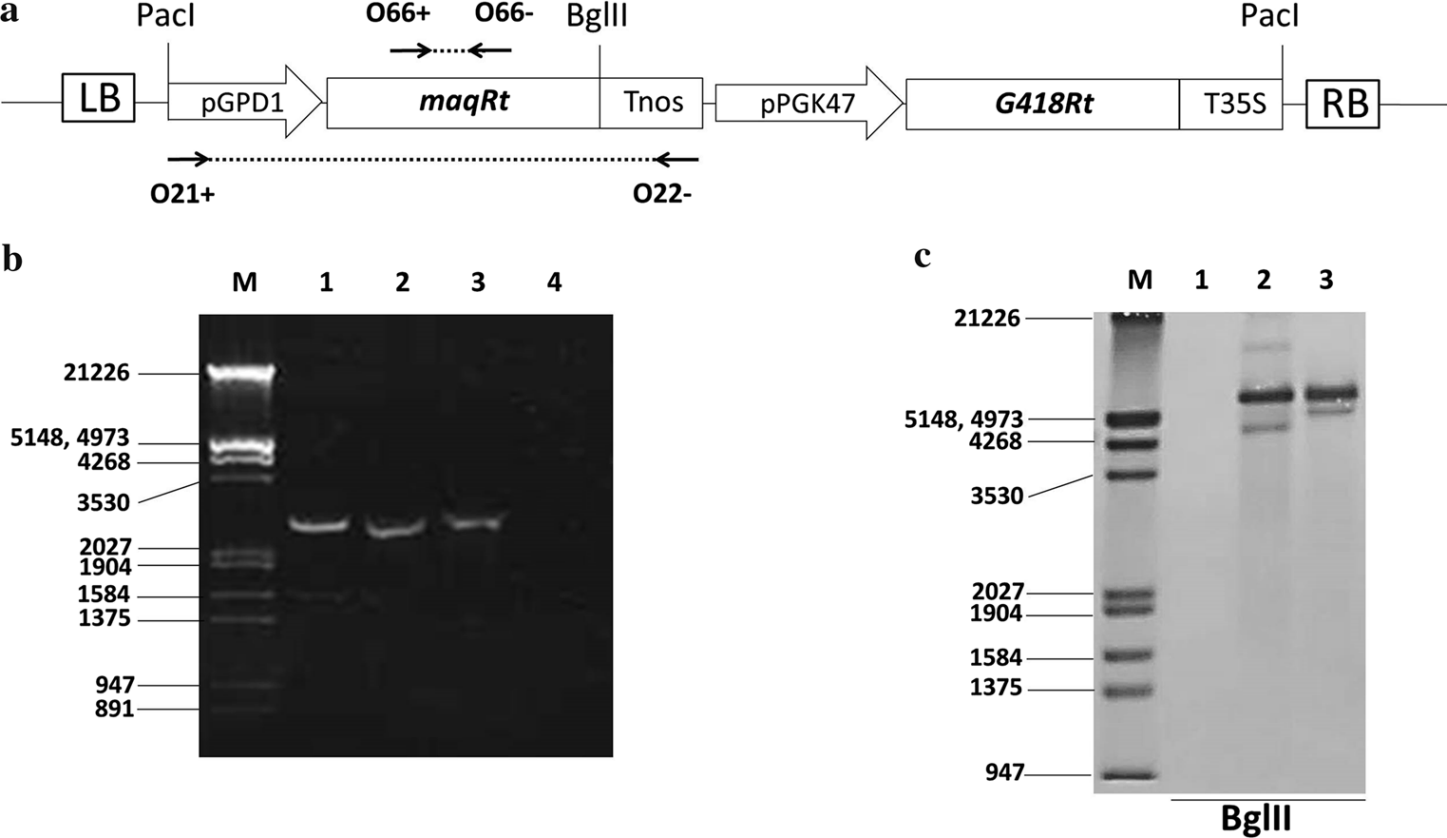
**a** Map of pNEOL102 containing the maqRt and G418Rt cassettes. *Black arrows* show the position of oligonucleotides O21+ and O22− used for PCR analysis, and oligonucleotides O66+ and O66− used for hybridization analysis (785 bp probe). *LB* T-DNA left border; *RB* T-DNA right border. **b** Amplification of maqRt cassette from genomic DNA. *M* molecular weight marker III; *1* *R. toruloides* CECT13085 clone #24; *2* *R. toruloides* CECT13085 NS-134 strain; *3* pNEOL102; *4* *R. toruloides* CECT13085 wild-type strain and **c** hybridization analysis. *M*, molecular weight marker III DIG-labeled; *1* *R. toruloides* CECT13085 wild-type strain; *2* clone #24; *3* NS-134 strain

Alcohol-forming FAR from *M. aquaeolei* VT8 Maqu_2220 (GenBank YP_959486.1, Supplementary Table 1) [15] was synthesized using *R. toruloides* codon. The resulting *maqRt* gene was cloned under control of glyceraldehyde 3-phosphate dehydrogenase promoter (pGPD1) amplified from *R. toruloides* CECT13085 genomic DNA [21]. We used Tnos, from the pBI101 plasmid, as terminator to obtain the maqRt cassette. The gene *G418Rt*, conferring resistance to Geneticin, was synthesized using *R. toruloides* codon (Supplementary Table 1) and cloned under control of phosphoglycerate kinase promoter (pPGK47) amplified from *R. toruloides* CECT13085 genomic DNA [19]. The T35S terminator from cauliflower mosaic virus 35S was synthesized and used as terminator to get the G418Rt cassette (Supplementary Table 1). The maqRt cassette was cloned next to the G418Rt cassette to yield pNEOL79. Finally, we cloned a 5 kb *Pac*I fragment from pNEOL79 into pNEOL57 to obtain pNEOL102 (Fig. 1a).

### *Rhodosporidium toruloides* transformation and clone selection

To integrate pNEOL102 in *R. toruloides* CECT13085, we used the *Agrobacterium tumefaciens*-mediated transformation method (ATMT) [17, 19, 21]. Briefly, *A. tumefaciens* carrying pNEOL102 was grown in an induction medium [5], whereas *R. toruloides* CECT 13085 was grown in YPD medium for 15 h. Strains were washed, incubated at 30 °C and 250 rpm for 6 h and co-cultivated for 3 days at 25 °C. The transformation mixture was plated on YPD medium supplemented with cefotaxime (200 µg/mL) and Geneticin (35 µg/mL), and plates were incubated at 30 °C for 48 h.

The clones were analyzed by PCR (Fig. 1a, b) using oligonucleotides O21+ (5′-ggactagtcgccgggatgccaacgtcgtt-3′) and O22− (5′-ccactagtaaatgtataattgcgggactc-3′). Integration of the expression cassette into *R. toruloides* CECT13085 genome was also confirmed by hybridization. We amplified a 785 kb DNA fragment from the *maqRt* gene with specific oligonucleotides O66+ (5′-gagatcgccacctcgtcggt-3′) and O66− (5′-agcgagaggatgatcgagtt-3′). Then we labeled it using a “DIG-High Prime DNA Labeling and Detection Starter Kit I” (Roche) and used it as a probe (Fig. 1a, c). Before performing hybridization, the genomic DNA of each strain was previously digested with *Bgl*II.

### Fatty alcohol extraction and analysis

Fatty alcohol production was analyzed both in the fermentation medium and inside the cells. Culture broth samples (50 mL) were centrifuged at 10,000×*g*/10 min. The broth was mixed with 30 mL of ethyl acetate three times and the organic phases were collected. An Na_2_SO_4_ anhydride solution was added to the organic phase and incubated for 30 min at room temperature with gentle agitation every 5–10 min. The sample was filtered and the solvent evaporated in a rotary evaporator until dry. Fatty alcohol extraction from cells was done according to the procedure described by Schneiter and Daum [31]. Namely, 2 g of cells was washed with 0.9 % sodium chloride, and methanol (13 mL) was added. Cellular lysis was achieved with the addition of 20 g of glass beads (0.25–0.3 mm of diameter) followed by a strong agitation (1000–1500 rpm, 1–10 min) and/or ultrasonic disruption (1–20 min). Then, 26 mL of chloroform was added and the mixture was placed in an orbital shaker at 150–200 rpm for 20–60 min and finally filtered. The organic phase was washed successively with magnesium chloride (0.034 %, w/v), 2 N potassium chloride:methanol solution (1:1, v/v) and a chloroform/methanol/water solution (3:48:7, v/v). After each wash, the two phases were separated by centrifugation at 3000×*g*/5 min and the light phases were removed and collected. Finally, this organic phase was evaporated in a rotary evaporator until dryness.

Fatty alcohol samples were derivatized with BSTFA (bis-trimethylsilyl-trifluoroacetamide, Sigma) and analyzed with a GC–MS Agilent 6890/5973 system equipped with a ZB-5 ms column (30 cm, 250 µm id, 0.25 µm thickness; Supelco). Helium was used as carrier gas with a constant flow of 0.8 mL/min. The oven temperature was 80 °C for 0.5 min and then increased (ramp rate 5 °C/min) to 250 °C for 5 min. Experimental conditions for mass spectrometer were: solvent delay (4 min), electron impact ionization (70 eV) and dwell time (100 ms). Chromatograms were registered by SCAN mode (mass range 50–500 *m/z*) and SIM mode (299.1/313.2/325.2/340.2/327.2/341.3 *m/z*). Fatty alcohol quantitation was done using calibration curves for oleyl, stearyl and cetyl alcohols, respectively.

### Seed and production media preparation for fermentation assays

Seed cultures in SC medium: sucrose 40 g/L (Azucarera, Spain) and corn steep liquor 9.6 g/L (Dadelos, Spain) were incubated at 30 °C and 250 rpm for 24 h. The production medium (PSC) was prepared using sucrose (100 g/L) supplemented with KH_2_PO_4_ 0.75 g/L, NH_4_NO_3_ 0.7 g/L, CaCl_2_·2H_2_O 0.4 g/L, MgSO_4_·7H_2_O 0.4 g/L, corn steep liquor 22.5 g/L and Tergitol™ L-81 5 g/L (Dow Chemical, USA). Medium pH was adjusted to pH 5.0 using 12.5 % NH_4_OH.

### Fermentation

Experiments were performed in a Biobundle ez-control bioreactor (Applikon^®^ Biotechnology, Delf, The Netherlands) equipped with a 7 L jacketed vessel. The production medium (1.8 L) was added to the reactor and sterilized in an autoclave (121 °C, 30 min). Once the medium was cooled down, culture conditions were set to 30 °C, pH 5 and 3 L/min aeration (1.5 vvm). The reactor was inoculated with 200 mL from a seed culture. The pH was maintained by adding 12.5 % NH_4_OH solution and *p*O_2_ was sustained above 30 % of air saturation by adopting the stirrer speed between 500 and 1000 rpm (agitation cascade). The sugar concentration in the broth was kept at 40–60 g/L by feeding sucrose (400 g/L). After 60 h of cultivation, sucrose feeding was stopped to achieve its full consumption.

### Fermentation analytical methods

Samples (70 mL) were taken every 12 h along the cultivation time. Twenty mL was used to determine growth (measure as dry cell weight, DCW). The sample was centrifuged (8000×*g*, 10 min) and the supernatant was used for sucrose analysis. The pellet was washed with 50 % ethanol and dried in an oven at 105 °C to constant weight. The remaining 50 mL was centrifuged (8500×*g*, 10 min.) and both the supernatant and the pellet were stored for fatty alcohol analysis.

Sucrose concentration in the fermentation broth was analyzed using a UPLC Acquity (Waters) with an ELSD Detector and an Acquity UPLC BEH Amide (100 mm × 2.1 mm × 1.7 µm film thickness, Waters) column. Microbial oil was extracted from a dried pellet by acidic hydrolysis (3 N HCl, 100 °C, 1 h) followed by filtration (Whatman 8 µm) and hexane extraction of the filter in a Soxhlet. Finally, hexane was evaporated and microbial oil quantified gravimetrically.

## Results

### Screening for fatty alcohol production

**Fig. 2:**
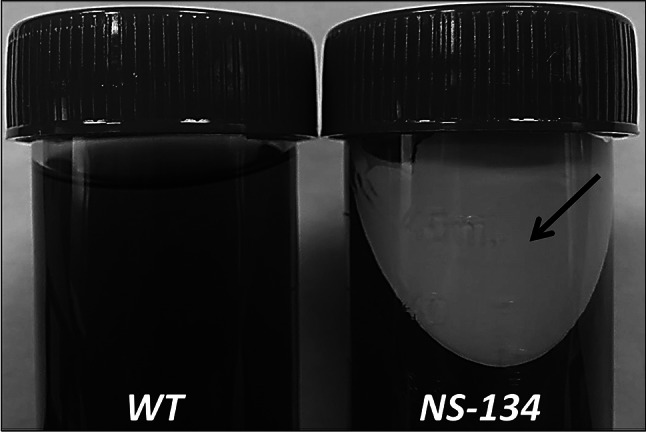
Screening for fatty alcohol production. *R. toruloides* CECT13085 wild-type strain (WT) and NS-134 strain after centrifugation (10 min, 10,000×*g*). The *black arrow* points to a white composition (fatty alcohol mixture) present in cultures of NS-134 strain

**Fig. 3:**
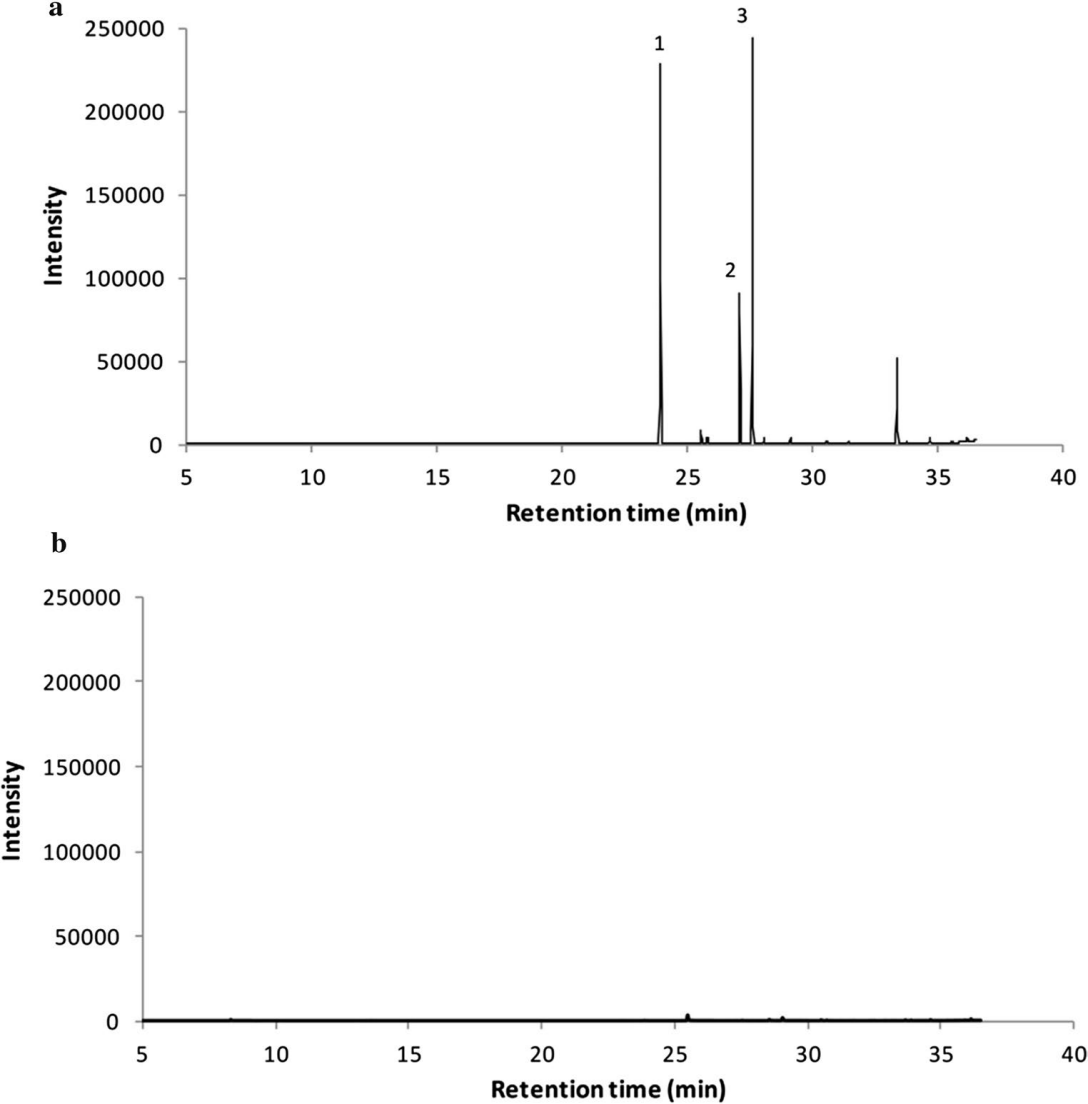
GC–MS analysis (SIM mode). **a** *R. toruloides* NS-134 strain. *1* Cetyl alcohol; *2* oleyl alcohol; *3* stearyl alcohol and **b** *R. toruloides* CECT13085 (wild type)

ATMT transformation efficiency in *R. toruloides* CECT13085 was ~1000 colonies per 10^7^ input cells, which is similar to previous results [19, 21]. After ATMT transformation, 30 clones able to grow on selection plates were randomly chosen to be analyzed by PCR to check the integration of maqRt cassette in *R. toruloides* CECT13085 genome. All clones showed the expected 2.4 kb band (Fig. 1b). We made a screening assay cultivating each clone in flasks containing the YPD medium. Interestingly, after 24 h of incubation, a white and insoluble extracellular compound was observed in several cultures, but not in the parental strain (Fig. 2). The best fatty alcohol producer, named NS-134, was analyzed by hybridization along with other clones and we observed ectopic integration of maqRt cassette in the genome (Fig. 1c). Two copies were present in the NS-134 genome, while clone 24 carried three copies. Fatty alcohols were extracted from the fermentation medium as described above and the resulting extract was analyzed by GC–MS. The SCAN mode chromatogram showed the presence of several peaks in the NS-134 strain (Supplementary Fig. 1). Using Wiley275 library, three mayor peaks were identified as cetyl, oleyl and stearyl alcohol with retention time (*t*_R_) of 23.91, 27.09 and 27.59 min, respectively. Two other peaks were identified as palmitic acid (*t*_R_: 25.55) and malonic acid (*t*_R_: 33.36). Other peaks were also present in the SCAN chromatogram, but we could not identify them, as their matching rates in the library were very low (lower than 40 %). Moreover, SCAN chromatogram of wild-type strain CECT13085 showed three free fatty acids identified as palmitic acid, stearic acid and oleic acid with a retention time (*t*_R_) of 25.48, 28.56 and 29.06 min, respectively, as well as other unknowns peaks (Supplementary Fig. 1). To achieve better specificity for fatty alcohols, SIM chromatograms (using *m/z* as described above) were registered (Fig. 3). The SIM mode chromatogram showed the presence of three major peaks in the selected clones, but absent in the wild-type strain. Those peaks, with a retention time of 24.09, 27.27 and 27.78 min, corresponded to cetyl, oleyl and stearyl alcohol, respectively (Fig. 3). A fourth peak corresponding to malonic acid (33.2 min) was also observed. The presence of malonic acid in extracts from *R. toruloides* NS-134 is not surprising, as it is a main precursor for fatty acid biosynthesis (fatty acyl-CoAs).

### Fatty alcohol production in different media

**Fig. 4:**
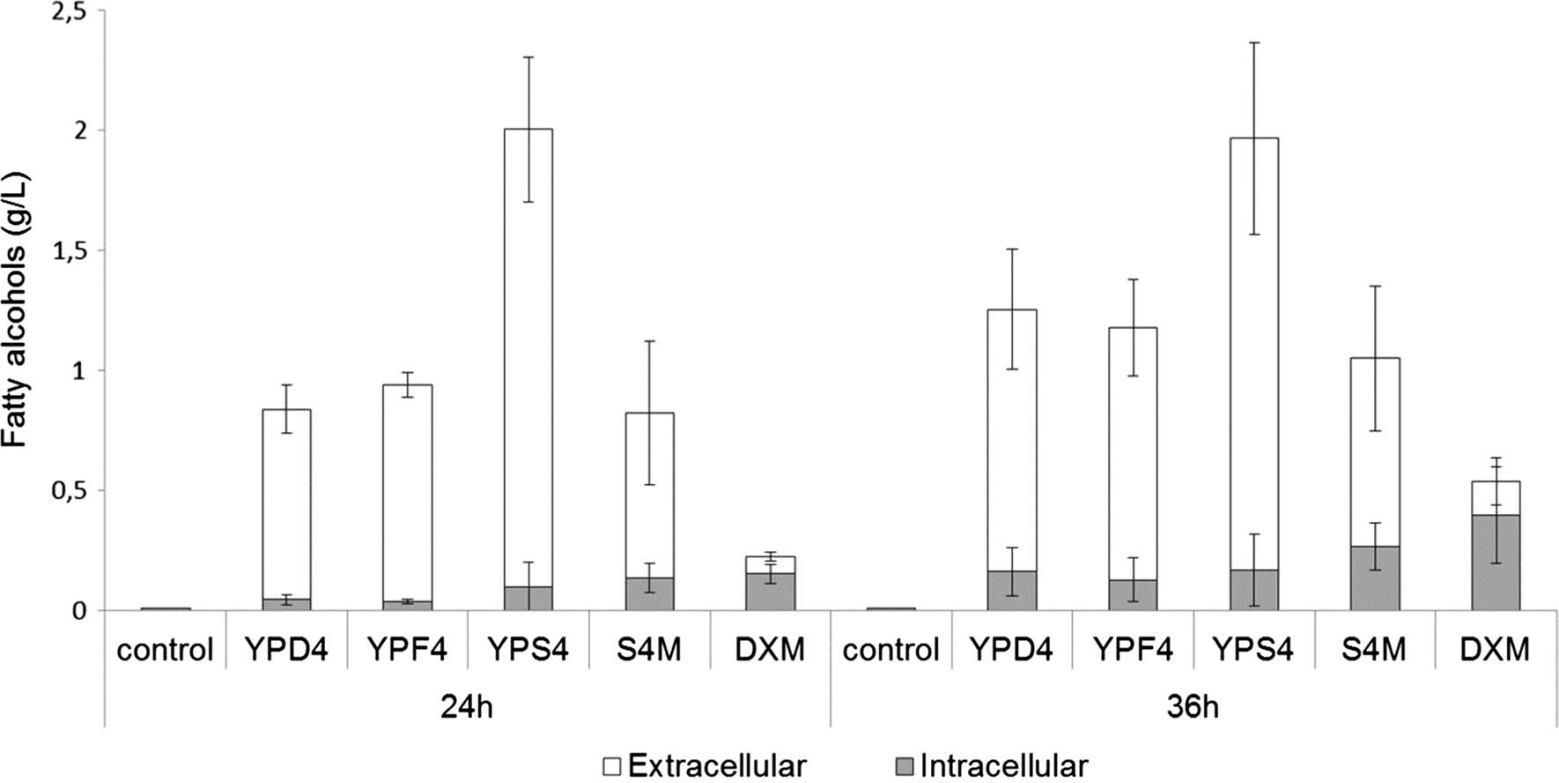
Fatty alcohol production in different media. Control: *R. toruloides* CECT13085 (wild-type) in YPD. The standard deviation was calculated from two independent experiments

The best fatty alcohol producer, NS-134 strain, was selected to analyze its capability to produce fatty alcohols in different fermentation media. As shown in Fig. 4, at 24 h, sucrose supported the highest titer (2 g/L), whereas with glucose or fructose it was about 0.8 or 0.9 g/L, respectively. After 36 h, fatty alcohol production remained the same for sucrose, whereas for glucose or fructose it increased up to 1.2 g/L. In all flasks, more than 85 % of the fatty alcohols were secreted into the fermentation media. The biomass production was very similar in all these media (about 3 g/L after 36 h). We also checked if the NS-134 strain was able to produce fatty alcohols in DXM medium containing a mixture of glucose and xylose. After 24 h of cultivation, NS-134 was able to produce 0.22 g/L of fatty alcohols in this medium (Fig. 4) and the titer increased almost twofold (0.53 g/L) at the end of the fermentation. However, unlike previous results, fatty alcohols were mainly intracellular. Indeed, about 75–80 % of the fatty alcohols were extracted from the yeast cells. These results were also observed with other media when fatty alcohol production was lower than 0.5 g/L (data not shown). Therefore, it seems that extracellular production is somehow related to the capability of a strain to produce fatty alcohols at a high titer (>1 g/L).

### High production of fatty alcohols in bioreactors

**Fig. 5:**
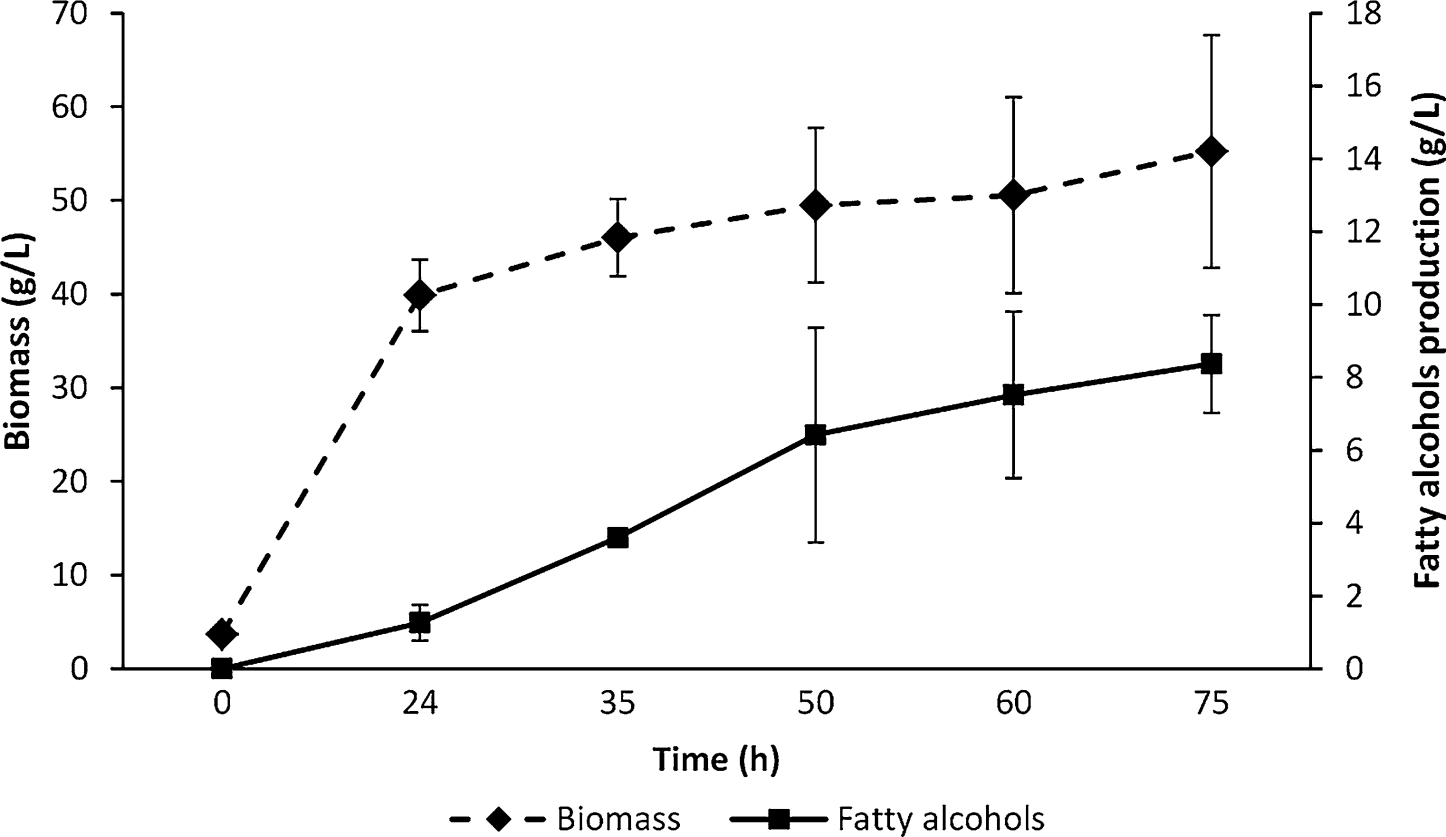
Cellular growth and fatty alcohol production in a 7 L bioreactor. The standard deviation was calculated from three independent fermentation assays

**Fig. 6:**
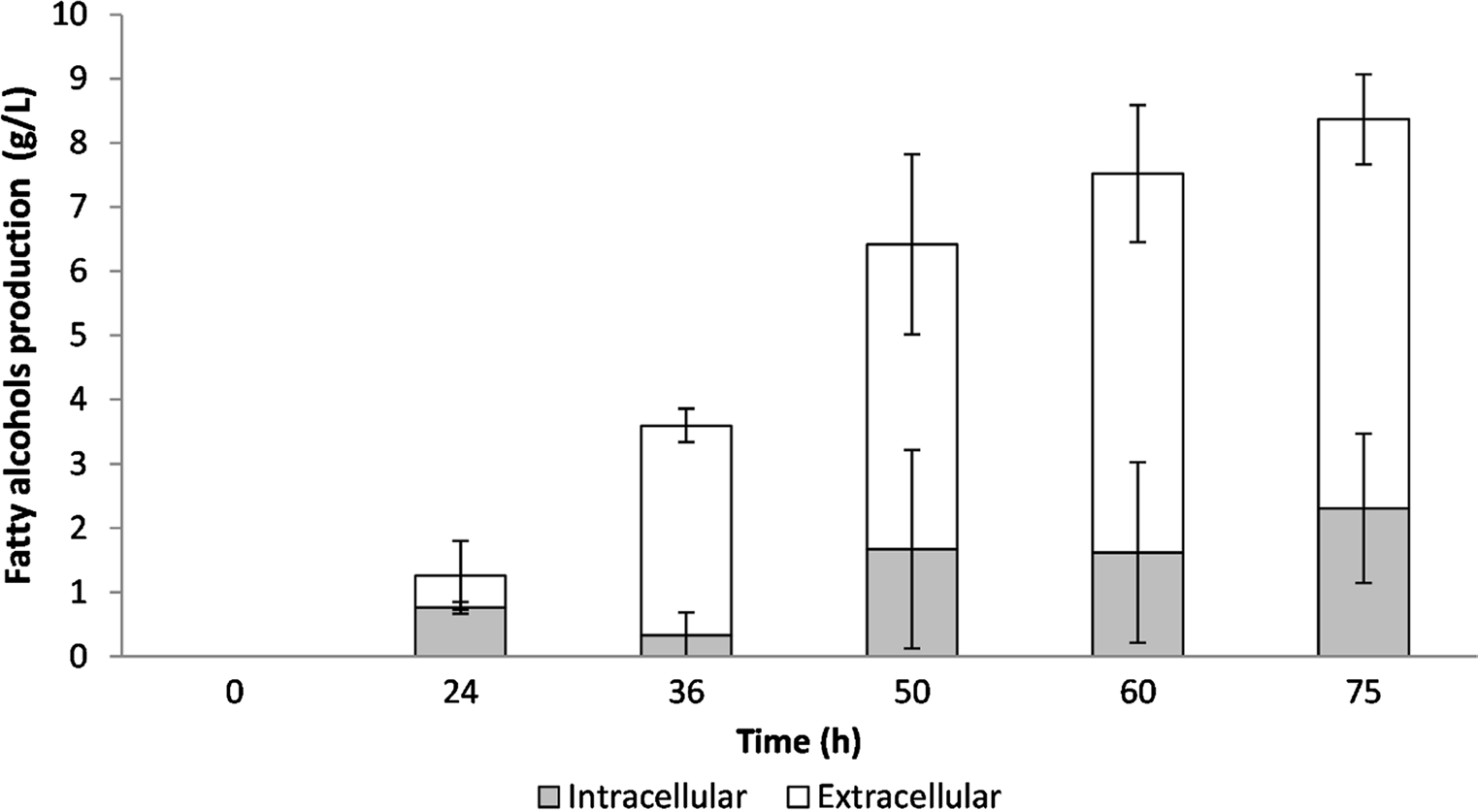
Intracellular and extracellular fatty alcohol distribution. Production was carried out in a 7 L bioreactor. The standard deviation was calculated from three independent fermentation assays

Based on the promising results obtained in the flasks, we carried out assays in a bioreactor using sucrose as carbon source. First, assays were carried out using a high carbon/nitrogen ratio (C/N = 20) to support microbial oil production (and fatty acyl-CoAs). Results showed a very high oil production (58 g/L), whereas fatty alcohol titer was quite low (<2 g/L, Table 2). Therefore, we reduced the carbon/nitrogen ratio (C/N = 3) by lowering CSL concentration (22.5 g/L) and using ammonia for pH control, to promote biomass production and reduce oil synthesis. Under this condition, cell biomass increased quite fast and reached 40 g/L after 24 h of cultivation. The growth rate lowered from this time to the end of fermentation (75 h) to obtain 55 g/L of the biomass (Fig. 5), while microbial oil production did not exceed 6.5 g/L (Table 2). Fatty alcohols were produced linearly along fermentation and reached over 8 g/L after 75 h of cultivation (Fig. 5). Longer incubation times did not improve fatty alcohol production and this actually decreased due to sucrose depletion. As it was previously reported in flasks, analysis of fatty alcohol distribution showed that about 72 % of the total fatty alcohols were located in the extracellular fraction (Fig. 6). GC–MS analysis revealed that fatty alcohols were 95 % of the extract, whereas the remaining 5 % was mainly made of free fatty acids. Oleyl alcohol was the most abundant (69 ± 1 %) followed by stearyl alcohol (19 ± 4 %) and cetyl alcohol (12 ± 3 %).

**Table 2:** Fatty alcohol (FA) production under different C/N ratios

C/N ratio	Time (h)	Biomass (g/L)	Oil (g/L)	Intracellular FAs (g/L)	Extracellular FAs (g/L)	Total FAs (g/L)	Productivity (g/L h)	Yield (g/g)
20	75	63.1	58.0	0.31	0.92	1.23	0.02	0.01
3	75	55.2	6.5	2.30	6.10	8.40	0.11	0.04

## Discussion

Oleaginous yeasts are considered attractive next-generation host candidates for the production of fatty acid-derived chemicals, as they have many advantages over *E. coli* and *S. cerevisiae*. Among those yeasts, *R. toruloides* is one of the best candidates based on its capability to produce high amounts of lipids [2, 3, 36] and use wide-scale renewable raw materials [21, 33, 34]. Furthermore, its genome has been sequenced [39], and efficient genetic transformation and targeted gene deletion methods have been recently reported [17, 21].

*Marinobacter aquaeolei* VT8 has two fatty acyl-CoA reductases able to directly reduce fatty acyl-CoA to the corresponding fatty alcohols without the fatty aldehyde intermediate [15, 34]. Heterologous expression of both genes (Maqu_2220 and Maqu_2507) has been used to produce up to 1.7 g/L fatty alcohols in *E. coli* after 104 h of batch cultivation in a 5 L fermenter [20].

Ectopic integration of codon-optimized *M. aquaeolei* VT8 Maqu_2220 gene into the genome of *R. toruloides* led to the production of over 8 g/L of fatty alcohols in fed-batch reactors. This simple modification in *R. toruloides* lipid metabolism pathway differs much from the complex and numerous changes made in *S. cerevisiae* or *E. coli* cells to produce fatty alcohols [12, 14, 28, 32, 33]. The use of this lipid-overproducing strain as host seems to provide all intermediates and substrates (e.g., fatty acyl-CoAs) needed to produce large amounts of different lipid derivatives including fatty alcohols.

Culture media for fatty alcohol production in *S. cerevisiae* or *E. coli* cells mainly contain glucose as carbon source [14, 20, 28]. However, fatty alcohol production in *S. cerevisiae* was improved using raffinose and galactose [33]. Because in *R. toruloides*, galactose metabolism is not very efficient (data not shown), we used glucose, fructose or sucrose as carbon source for the production of fatty alcohols. In flasks, our results clearly showed that sucrose supported the highest fatty alcohol production (Fig. 4). This result is more likely due to the good production achieved both in glucose and fructose, and could also be a matter of a slower release of both carbohydrates from the sucrose unit. Interestingly, production was also achieved in DXM medium. The formulation of this medium resembles the composition of a lignocellulosic hydrolysate as it contains a mixture of sugars (glucose and xylose) as well as organic acids (acetic and formic) and furfural. Although production in this medium was much lower than that obtained with other media, these results indicate the potential use of lignocellulosic biomass hydrolysates as raw material for the production of fatty alcohols with this strain.

*Saccharomyces cerevisiae* seems to secrete fatty alcohols into the culture medium as they were recovered from a dodecane overlay used to reduce their evaporation [12, 28]. However, Tang and Chen [33] reported that only a small amount of fatty alcohols (about 10 % of the total) was found in the fermentation medium, whereas the remaining 90 % were intracellular. According to our results, fatty alcohols produced by *R. toruloides* were mainly extracellular (>72 % of the total) (Fig. 6) under almost all conditions tested. This fact could be a key competitive advantage for the development of an industrial process to produce fatty alcohols by fermentation. Only in DXM, medium production seemed to be mainly intracellular; however, this issue could be probably due to the low fatty alcohol production since it was also observed in other medium (data not shown).

In *S. cerevisiae* cells, cetyl alcohol (C16:0) was found to be the most abundant, representing about 80–90 % of the total fatty alcohols, whereas stearyl alcohol (C18:0) accounted for 8–10 % [33]. Analysis of fatty alcohols produced by *R. toruloides* NS-134 showed a composition quite different from oleyl alcohol (C18:1), stearyl alcohol (C18:0) and cetyl alcohol (C16:0), representing 57, 24 and 19 % of the total fatty alcohols (intra- and extracellular fractions), respectively. Such differences are more likely due to the fatty acid composition of the oil produced by this oleaginous strain (60 % oleic acid, 23 % palmitic acid, 11 % stearic acid and 6 % linoleic acid) and to the broad substrate specificity of *M. aquaeolei* VT8 Maqu_2220 reductase [15, 34].

Although we have achieved the highest titer ever reported (8 g/L), the yield of fatty alcohols (0.04 g/g sucrose) is far away from the theoretical yield (0.33 g/g sucrose). This means that there are some rate-limiting steps that must be overcome to improve fatty alcohol production. One possible way to improve such production could be to increase the intracellular pool of fatty acyl-CoAs, the substrate for the fatty alcohols forming reductases.

In yeast cells, fatty acyl-CoAs released from the FAS complex are either used for the synthesis of steryl ester or destined for the triglycerides (TAG) storage pathway where *DGA1* and *LRO1* genes play a predominant role [9, 16, 29]. Disruption of the *DGA1* and *LRO1* genes did not compromise the viability of *S. cerevisiae* cells, but reduced the turnover of fatty acyl-CoAs to TAG [29]. Consistent with these observations, the disruption of *DGA1* gene increased 1.5-fold the production of fatty alcohols in *S. cerevisiae* [33].

Genomic data analysis from our strain (Fillet et al., unpublished results) and *R. toruloides* NP11 [39] confirmed that enzymes involved in lipid synthesis in *R. toruloides* are similar to those found in *S. cerevisiae* except for the presence of ATP:citrate lyase (ACL), which catalyzes the production of acetyl-CoA by cleavage of citrate in oleaginous species [27].

Based on the above information, we are currently trying to engineer the lipid metabolic pathway of *R. toruloides* NS-134 by knocking out *DGA1* and/or *LRO1* genes to improve the intracellular pool of fatty acyl-CoAs in this yeast.

## Electronic supplementary material

Supplementary material 1 (DOCX 126 kb)
