# Supplementary material for: Fatty alcohols production by oleaginous yeast
Source: J Ind Microbiol Biotechnol. 2015 Aug 29;42(11):1463–72. doi: 10.1007/s10295-015-1674-x (PMC4607723; doi:10.1007/s10295-015-1674-x)
Supplement: Supplementary file 1 — Supplementary material 1 (DOCX 126 kb) [file 10295_2015_1674_MOESM1_ESM.docx]

**Supplementary material**

**Supplementary Table 1**. Synthetized genes designed with *R. toruloides* codon usage (http://www.bioinformatics.org/sms2/rev_trans.html)

| **Gene** | **GenBank accession number** | **Nucleotide sequence** |
| --- | --- | --- |
| *MaqRt* | YP_959486.1 | atggccatccagcaggtccaccacgccgacacctcgtcgtcgaaggtcctcggccagctccgcggcaagcgcgtcctcatcaccggcaccaccggcttcctcggcaaggtcgtcctcgagcgcctcatccgcgccgtcccggacatcggcgccatctacctcctcatccgcggcaacaagcgccacccggacgcccgctcgcgcttcctcgaggagatcgccacctcgtcggtcttcgaccgcctccgcgaggccgactcggagggcttcgacgccttcctcgaggagcgcatccactgcgtcaccggcgaggtcaccgaggccggcttcggcatcggccaggaggactaccgcaagctcgccaccgagctcgacgccgtcatcaactcggccgcctcggtcaacttccgcgaggagctcgacaaggccctcgccatcaacaccctctgcctccgcaacatcgccggcatggtcgacctcaacccgaagctcgccgtcctccaggtctcgacctgctacgtcaacggcatgaactcgggccaggtcaccgagtcggtcatcaagccggccggcgaggccgtcccgcgctcgccggacggcttctacgagatcgaggagctcgtccgcctcctccaggacaagatcgaggacgtccaggcccgctactcgggcaaggtcctcgagcgcaagctcgtcgacctcggcatccgcgaggccaaccgctacggctggtcggacacctacaccttcaccaagtggctcggcgagcagctcctcatgaaggccctcaacggccgcaccctcaccatcctccgcccgtcgatcatcgagtcggccctcgaggagccggccccgggctggatcgagggcgtcaaggtcgccgacgccatcatcctcgcctacgcccgcgagaaggtcaccctcttcccgggcaagcgctcgggcatcatcgacgtcatcccggtcgacctcgtcgccaactcgatcatcctctcgctcgccgaggccctcggcgagccgggccgccgccgcatctaccagtgctgctcgggcggcggcaacccgatctcgctcggcgagttcatcgaccacctcatggccgagtcgaaggccaactacgccgcctacgaccacctcttctaccgccagccgtcgaagccgttcctcgccgtcaaccgcgccctcttcgacctcgtcatctcgggcgtccgcctcccgctctcgctcaccgaccgcgtcctcaagctcctcggcaactcgcgcgacctcaagatgctccgcaacctcgacaccacccagtcgctcgccaccatcttcggcttctacaccgccccggactacatcttccgcaacgacgagctcatggccctcgccaaccgcatgggcgaggtcgacaagggcctcttcccggtcgacgcccgcctcatcgactgggagctctacctccgcaagatccacctcgccggcctcaaccgctacgccctcaaggagcgcaaggtctactcgctcaagaccgcccgccagcgcaagaaggccgcctag |
| *G418Rt* | AF195002.1 | atgggcaaggagaagacccacgtctcgcgcccgcgcctcaactcgaacatggacgccgacctctacggctacaagtgggcccgcgacaacgtcggccagtcgggcgccaccatctaccgcctctacggcaagccggacgccccggagctcttcctcaagcacggcaagggctcggtcgccaacgacgtcaccgacgagatggtccgcctcaactggctcaccgagttcatgccgctcccgaccatcaagcacttcatccgcaccccggacgacgcctggctcctcaccaccgccatcccgggcaagaccgccttccaggtcctcgaggagtacccggactcgggcgagaacatcgtcgacgccctcgccgtcttcctccgccgcctccactcgatcccggtctgcaactgcccgttcaactcggaccgcgtcttccgcctcgcccaggcccagtcgcgcatgaacaacggcctcgtcgacgcctcggacttcgacgacgagcgcaacggctggccggtcgagcaggtctggaaggagatgcacaagctcctcccgttctcgccggactcggtcgtcacccacggcgacttctcgctcgacaacctcatcttcgacgagggcaagctcatcggctgcatcgacgtcggccgcgtcggcatcgccgaccgctaccaggacctcgccatcctctggaactgcctcggcgagttctcgccgtcgctccagaagcgcctcttccagaagtacggcatcgacaacccggacatgaacaagctccagttccacctcatgctcgacgagttcttctag |
| *T35S* | KJ716236.1 | acgctgaaatcaccagtctctctCtacaaatctatctctctctattttctccataaataatgtgtgagtagtttcccgataagggaaaTtagggttcttatagggtttcgctcatgtgttgagcatataagaaacccttagtatgtatttgtatttgtaaaatacttctatcaataaaatttctaattcctaaaaccaaaatccagtactaaaatccaga |


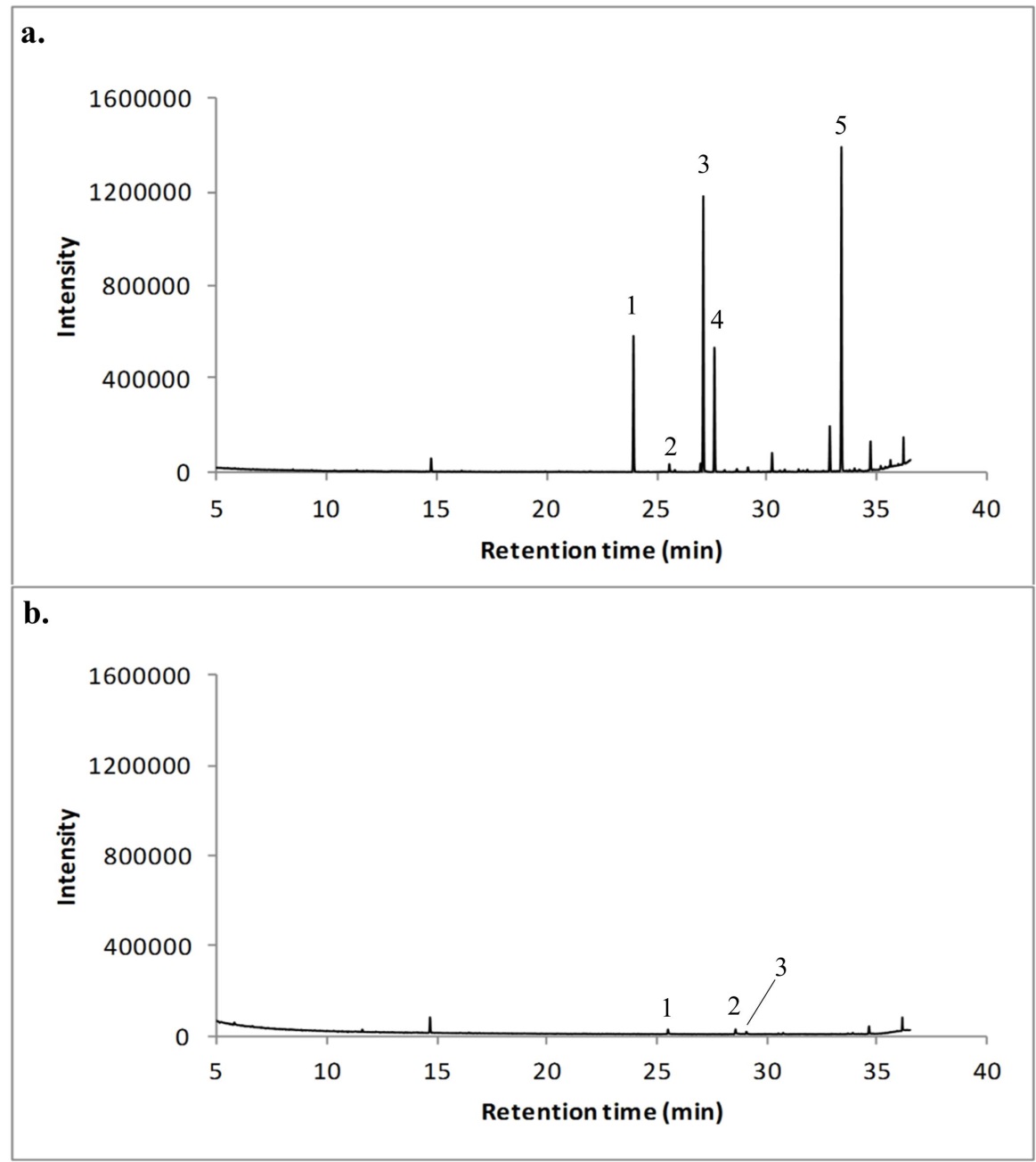
**Supplementary Fig. 1**. GC-MS analysis (SCAN mode). a: *R. toruloides* NS-134 strain. 1: cetyl alcohol; 2: palmitic acid; 3: oleyl alcohol; 4: stearyl alcohol; 5: malonic acid. b: *R. toruloides* CECT13085 (wild type). 1: palmitic acid; 2: stearic acid; 3: oleic acid.
